# Predictors of stillbirth among women who had given birth in Southern Ethiopia, 2020: A case-control study

**DOI:** 10.1371/journal.pone.0249865

**Published:** 2021-05-03

**Authors:** Haimanot Abebe, Solomon Shitu, Haile Workye, Ayenew Mose

**Affiliations:** 1 Department of Public Health, College of Health Sciences and Medicine, Wolkite University, Wolkite, Ethiopia; 2 Department of Midwifery, College of Health Sciences and Medicine, Wolkite University, Wolkite, Ethiopia; 3 Department of Nursing, College of Health Sciences and Medicine, Wolkite University, Wolkite, Ethiopia; South China Agricultural University, CHINA

## Abstract

**Background:**

Although the rate of stillbirth has decreased globally, it remains unacceptably high in low- and middle-income countries. Only ten countries including Ethiopia attribute more than 65% of global burden of still birth. Ethiopia has the 7^th^ highest still birth rate in the world. Identifying the predictors of stillbirth is critical for developing successful interventions and monitoring public health programs. Although certain studies have assessed the predictors of stillbirth, they failed in identify the proximate predictors of stillbirth. In addition, the inconsistent findings in identify the predictors of stillbirth, and the methodological limitations in previously published works are some of the gaps. Therefore, this study aimed to identify the predictors of stillbirth among mothers who gave birth in six referral hospitals in Southern, Ethiopia.

**Methods:**

A hospital-based unmatched case-control study was conducted in six referral hospitals in Southern, Ethiopia from October 2019 to June 2020. Consecutive sampling techniques and simple random techniques were used to recruit cases and controls respectively. A structured standard tool was used to identify the predictors of stillbirth. Data were entered into Epi Info 7 and exported to SPSS 23 for analysis. A multivariable logistic regression model was used to identify the independent predictors of stillbirth. The goodness of fit was tested using the Hosmer and Lemeshow goodness-of-fit. In this study P-value < 0.05 was considered to declare a result as a statistically significant association.

**Results:**

In this study 138 stillbirth cases and 269 controls were included. Women with multiple pregnancy [AOR = 2.98, 95%CI: 1.39–6.36], having preterm birth [AOR = 2.83, 95%CI: 1.58–508], having cesarean mode of delivery [AOR = 3.19, 95%CI: 1.87–5.44], having no ANC visit [AOR = 4.17, 95%CI: 2.38–7.33], and being hypertensive during pregnancy [AOR = 3.43, 95%CI: 1.93–6.06] were significantly associated with stillbirth.

**Conclusions:**

The predictors of stillbirth identified are manageable and can be amenable to interventions. Therefore, strengthening maternal antenatal care utilization should be encouraged by providing appropriate information to the mothers. There is a need to identify, screen, and critically follow high-risk mothers: those who have different complications during pregnancy, and those undergoing cesarean section due to different indications.

## Introduction

Stillbirth is a global public health problem, especially in Sub- Saharan Africa (SSA) and South Asia [1–4]. The WHO explained stillbirth as fetal death (i.e., death before the complete expulsion or extraction of a product of conception from its mother) in the third trimester (≥28 completed weeks of development) or with birth weight ≥1000 g or length ≥35 cm [5–7]. It is also an indicator of the quality of care during pregnancy and childbirth; institutional deliveries are increasing but stillbirth is not well decreased in the developing world [8–11].

Globally, there were 2.6 million stillbirths, of which the majority of deaths occurred in developing countries [7]. Ninety-eight percent occurred in low- and middle-income countries including South Asia and Sub-Saharan Africa [7, 8, 12]. The stillbirth rate in sub-Saharan Africa is approximately 10 times that of developed countries (29 vs 0. 3 per 1000 births) [7, 12]. An observational multi-country study from sub-Saharan Africa showed that the magnitude of stillbirth ranges from 28.9–154.6% per 1000 [13–17]. Hence, stillbirth rates are unacceptably high in a low-middle income country.

Previous researchers have revealed that asphyxia, maternal infection, non-communicable disease, chronic illness, resident, interpregnancy interval, previous preterm birth, premature rupture of membrane, the induced onset of labor, prolonged labor (>12 hours), multiple pregnancies, mode of delivery, maternal age, place of residence, education level, parity, antenatal care utilization, place of delivery, body mass index (BMI) and anemia, previous stillbirth, uterine rupture, abruption placentae, belonging to the poorest family, antepartum hemorrhage, maternal hypertensive disorder during pregnancy, and small weight-for gestational age babies are significant predictors of stillbirth [13–29].

The stillbirth rate is an important indicator of the quality of care during pregnancy and childbirth, as well as a sensitive marker of the health-care system. Post-2015 initiatives show that stillbirths are a hidden agenda worldwide and continue to be a sustainable development goal. A target to end preventable stillbirths was included in the Every Newborn Action Plan and endorsed for 194 countries including Ethiopia on the world health assembly in 2014. The plan was set with the goal of reducing the national stillbirth rate to 12 or fewer per 1000 births by 2030 [30]. Identifying predictors of stillbirth would contribute to the realization of a global target of stillbirth reduction in one or another way.

Ethiopia is among the countries with the highest stillbirth rate worldwide [12, 30]. According to the 2016 and 2019 Ethiopian Demographic and Health Survey (EDHS) report, the stillbirth ratio was 11 and 12 per 1000 live births [31, 32]. Stillbirth is an adverse birth outcome and represents a major problem in developing countries including Ethiopia. To curb this high stillbirth rate and neonatal morbidity, identifying the predictors of stillbirth is critical for developing successful interventions and monitoring public health programs. Although certain studies have assessed the predictors of stillbirth, they failed in identify the proximate predictors of stillbirth. Besides, the inconsistent findings of the predictors of stillbirth, and the methodological limitations in previously published works are some of the gaps. Therefore, this study aimed to identify the predictors of stillbirth among mothers who had given birth in six referral hospitals in Southern, Ethiopia.

In addition to, the main importance of this study for public health is: identify the potential predictors that predispose the fetus to stillbirth conditions is very important to tackle the underlying causes and to provide immediate solutions. The findings of this study initiate different stakeholders in the health care system to design appropriate strategies and planning for the measurements to be taken to avoid those potential factors, both in health care institutions as well as in the community at large. This study is an input for health policymakers and program developers typical of neonatal and child health in the health care delivery system.

## Methods and materials

### Study area

The southern region is an administrative region of Ethiopia. It has 23 zones and seven special woreda. Hawassa is the capital city of the Southern region. It is found 270 km southeast of Addis Ababa, the capital of Ethiopia. According to the 2007 national household census, the region has a total population of 14,929,548, of whom 7,425,918 were men and 7,503,630 women [33]. Six specialized hospitals serve the entire population in the region. All hospitals in the region provide comprehensive emergency obstetric care services. Additionally, 1689 health centers provide basic emergency obstetric care services in the Southern region. All six hospitals in the Southern region were involved in the study.

### Study period

The study was conducted from October 2019 to June 2020 among mothers who had given birth in six referral hospitals in Southern, Ethiopia.

### Study design

A hospital-based unmatched case-control study was conducted among mothers who had given birth in six referral hospitals of Southern, Ethiopia.

### Study population

All mothers who had given birth at six referral hospitals were included in the study. Case: is defined as fetal death after 28 weeks of pregnancy (either pre-partum or intrapartum stillbirth) and Control: is defined as live births after 28 weeks of pregnancy. Gestational age was determined using the obstetric ultrasonography report.

### Inclusion and exclusion criteria

Mothers who had given still and live births after 28 weeks of gestation during the study period were eligible for cases and controls respectively. Live and stillbirths with maternal mortality and mothers who were not permanent residents of the Southern region (lived for less than six months in the study area) were excluded.

### Sample size determination

The sample size was determined using the Epi Info 7 software StatCalc menu for an unmatched case-control study. The following assumptions were considered to calculate the sample size: a power of 80%, a confidence level of 95%, and a control to case ratio of (2:1). The proportion of stillbirths among mothers who had a clear color of the liquor was 28.7% with therefore the odds of developing stillbirth among mothers who had the green or light brown color of liquor during delivery was 2.0 times higher compared to women who had a clear color of liquor [20]. The total sample size required was 413 women (138 cases and 275 controls).

### Sampling technique and procedure

A consecutive sampling technique was used to select cases and a simple random sampling technique was used to recruit controls. Cases were identified using hospital admission logbooks, operation theater logbooks, and patient cards by Midwives and/or nurses in the respective hospitals. Two controls were selected using a simple random sampling technique among eligible mothers.

### Data collection tool and procedure

The tool was developed after exhaustively reviewing relevant literature [13, 16, 20, 23–28]. A minor modification was made to suit the local context. A pre-tested, structured interviewer-administered questionnaire and standard abstraction checklist to review data from medical records was used to collect the data. The questionnaire comprises socioeconomic characteristics, information on maternal and child health services. Stillbirth events were identified by midwives, and nurses in the obstetrics and gynecology wards. The data collectors were junior midwives and nurses. The data collection process was supervised by three trained general practitioners working in the study hospitals. Mothers were informed about the small print of the research before they provided consent to participate. Data were collected by interviewing mothers at admission or later during their hospital occupation. Both interviewing and data extraction from patient records was performed using similar data collectors.

### Operational definitions

#### Stillbirth

According to the World Health Organization (WHO) definition, stillbirth was defined as late fetal death with ≥ 28 completed weeks of gestation. Recent estimates of stillbirths published within the lancet were supported by an equivalent operational definition [34].

#### Gestational age

It was estimated based on an obstetric ultrasound report [14].

#### Preterm

A baby born at less than 37 weeks of gestation was considered to be pre-term [14].

### Data quality measures

To ensure the quality of the data (to validate the questionnaire before testing on study participants) the English language questionnaire was translated into the Amharic language (a language spoken in the study area) by an Amharic language speaker who has attended the Master of Arts in Amharic language and was translated back to the English language by a person who attended Master of Arts in English language and comparison was made on the consistency of the two versions. Three days of training were given to data collectors and supervisors about techniques of data collection, data abstraction template, the objective of the study, and briefed on each question included in the data collection tool. After the training was given, a pre-test was conducted a week before the actual survey in a comparable hospital on 5% of the sample size to ensure the validity of the tool, then correction was made. Proper coding and categorization of data were maintained for the quality of the data to be analyzed. The principal investigator and supervisors were frequently checked for consistency, accuracy, clarity, and completeness of the collected data and appropriate corrections were made on the spot.

### Data processing and analysis

Data were coded and entered into Epi Info7 and then exported to SPSS version 23 for analysis. The data were cleaned prior to analysis. Descriptive statistics such as frequencies, proportions, medians, and means were used to explain important variables to the outcome variable. The chi-square test was used to compare the proportion of cases and controls in terms of selected categorical variables. An independent sample t-test was also used to test the equality of means for selected continuous variables among cases and controls.

Bivariate analysis was performed using a binary logistic regression model to determine the association between each independent variable and stillbirth cases. The goodness of fit was tested using the Hosmer-Lemeshow goodness of fit test and Omnibus tests. To include the variables in the final model P < 0.2 in the bivariate analysis, and context point of view were considered. A multivariate logistic regression model was used to ascertain the independent effects. Multi-collinearity was checked using collinearity statistics (Variance inflation factor >10 and standard error >2). A crude and adjusted odds ratio (OR) with 95%CI was estimated to identify predictors of stillbirth. In this study P-value < 0.05 was considered to declare a result as a statistically significant association.

### Ethical approval and consent to participate

Ethical clearance was obtained from the Wolkite University College of Health and Medical Science Institutional Health Research Ethical Review Committee. An official letter was sent to the Sothern health bureau and the data collection was begun after permission and a cooperation letter was written to all referral hospitals on which the study was carried out. The study, purpose, procedure, duration, rights of the respondents, and data safety issues, possible risks, and benefits of the study were clearly explained to each participant using the local language. Furthermore, a one-page written summary of study information was given to those women who can read and understand the Amharic language. All subjects provided informed written consent for inclusion before they participated in the study.

## Results

### Socio-demographic characteristics of the mother

In this study, 138 cases and 269 controls were included with a response rate of 98.5% for both cases, and controls. The mean age of the case and controls was 28.1 and 27.2 years respectively. However, the mean age difference between the case and control groups was not statistically significant in the independent sample t-test (p = 0.23). Nearly all, 135 (97.8%) of the cases and 264 (98.1%) of the controls were married. Almost three-fifths, 113 (76.2%) of the cases and 205 (78.1%) of the controls were rural by residence. Regarding the educational status of the mother, 74 (53.6%) of the cases and 135 (50.2%) of the controls did not attend formal education. Nearly two-thirds, 90(65.2%) of the cases and 186(69.1%) of the controls were housewives by occupation. Other socio-demographic characteristics of the mothers are summarized in Table 1.

**Table 1:** Socio-demographic characteristics of mothers who had given birth in South Ethiopia, 2020 (N = 407).

Variable	Category	Cases (n = 138)		Controls (n = 269)	
		Frequency	Percentage (%)	Frequency	Percentage (%)
Residence	Rural	113	76.2	205	78.1
	Urban	25	23.8	45	21.9
Religion	Orthodox	63	45.7	169	62.8
	Muslim	28	20.3	64	23.8
	Protestant	47	34.1	36	13.4
Marital status	Married	135	97.8	264	98.1
	Divorced	3	2.2	5	1.9
Maternal occupation	Housewife	90	65.2	186	69.1
	Merchant	27	19.6	32	11.9
	Employed	20	14.5	42	15.6
	Farmer	1	0.7	9	3.3
Education status of women	No formal Education	74	53.6	135	50.2
	Primary school Education	46	33.3	82	30.5
	Secondary school Education and above	18	13.0	52	19.3

### Obstetrics history of mothers

**Fig 1:**
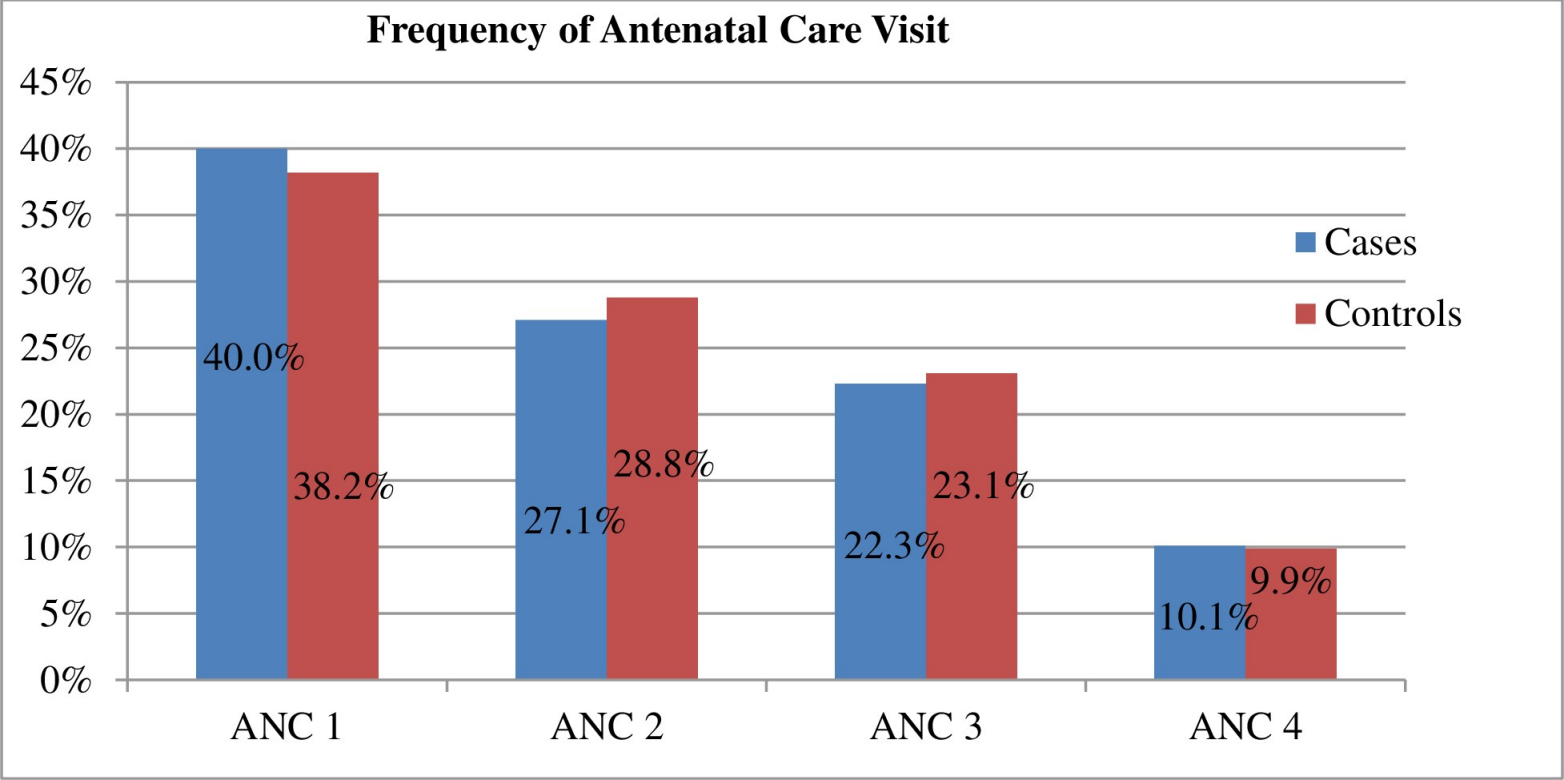
Frequency of ANC visit among mothers who had given birth in South Ethiopia, 2020 (N = 407).

Regarding antenatal care, 13.4% of mothers of controls had no antenatal care visits during the last pregnancy while 38.4% of mothers of cases had no antenatal care visits during the last pregnancy. The difference in antenatal care among mothers of cases and controls was statistically significant with the chi-square test **(P-value = 0.001**) (See Fig 1).

Fifteen (10.9%) mothers of cases had a history of abortion while 8.9% of mothers of controls had a history of abortion. The difference was not statistically significant in the chi-square test (***P-value = 0*.*072***).

Concerning parity, (22.5%) of mothers of the cases were grand multi-parous, followed by multi-parous (60.9%) and primiparous (16.7%). Forty-four (31.9%) mothers of cases had hypertension during pregnancy while 15.6% of mothers of controls had hypertension during pregnancy. The difference in hypertension during pregnancy among mothers of cases and controls was statistically significant with the chi-square test **(P-value = 0.003**). Other obstetric histories of the cases and controls are summarized in Table 2.

**Table 2:** Obstetrics history of mothers who had given birth in South Ethiopia, 2020 (N = 407).

Variable	Category	Cases (n = 138)		Controls (n = 269)	
		Frequency	Percentage (%)	Frequency	Percentage (%)
ANC visit	NO	53	38.4	36	13.4
	Yes	85	61.6	233	86.6
History of Abortion	Yes	15	10.9	24	8.9
	No	123	89.1	245	91.1
Hypertension during Pregnancy	Yes	44	31.9	42	15.6
	No	94	68.1	227	84.4
Mode of delivery	Cesarean section	60	43.5	47	17.5
	Vaginal	78	56.5	222	82.5
Parity	Primiparous	23	16.7	47	17.5
	Multiparous	84	60.9	159	59.1
	Grand Multiparous	31	22.5	63	23.4
Multiple pregnancies	Yes	27	19.6	15	5.6
	No	111	80.4	254	94.4
PROM	Yes	31	22.5	45	16.7
	No	107	77.5	224	83.3
Presence of HIV	Yes	24	17.4	34	12.6
	No	114	82.6	235	87.4
Length of Labor	≥24 hrs	66	47.8	110	40.9
	<24hr	72	52.2	159	59.1
Gestational Age	Preterm birth	46	33.3	32	11.9
	Term birth	92	66.7	237	88.1

### Predictors of stillbirth

Multivariable logistic regression model identified significantly associated factors with still birth which includes, women with multiple pregnancy [AOR = 2.98, 95%CI: 1.39–6.36], having preterm birth [AOR = 2.83, 95%CI: 1.58–508], having cesarean mode of delivery [AOR = 3.19, 95%CI: 1.87–5.44], having no ANC visit [AOR = 4.17, 95%CI: 2.38–7.33], and being hypertensive during pregnancy [AOR = 3.43, 95%CI: 1.93–6.06] were significantly associated with stillbirth.

The odds of stillbirth were 4.2 times higher among mothers who had no ANC visits during pregnancy compared to mothers who had no ANC visit. Mothers who had hypertension during pregnancy had 3.4 times higher odds of stillbirth compared to mothers who had no hypertension during pregnancy.

Cesarean section is a significant predictor of stillbirth. Mothers who had delivered in the cesarean section had 3.2 times higher odds of stillbirth compared to mothers who had delivered by vaginal mode of delivery. The overall model fitness (Hosmer-Lemeshow) and Omnibus test showed that the model fitted the data with a p-value of 0.883 and p-value of< 0.001 respectively. The predictors of stillbirth are summarized in Table 3.

**Table 3:** Multivariable logistic regression model for predictors of stillbirth among mothers who had given birth in South Ethiopia, 2020 (N = 407).

Variable	Category	Cases [N = 138]	Controls [269]	COR, 95% CI	AOR, 95%CI	P-value
Residency	Rural	113	205	1.41(0.84–2.36)	1.29(0.71–2.34)	0.39
	Urban	25	64	1	1	
Educational Status	No formal Education	74	135	1.58(0.86–2.90)	1.46(0.71–2.98)	0.30
	Primary Education	46	82	1.62(0.85–3.09)	1.77(0.83–3.78)	0.14
	Secondary Education and above	18	52	1	1	
ANC visit	No	53	36	4.04(2.47–6.59)	4.17(2.38–7.33)*	<0.001
	Yes	85	233	1	1	
Hypertension during Pregnancy	Yes	44	42	2.53(1.56–4.12)	3.43(1.93–6.06)*	<0.001
	No	94	227	1		
Mode of delivery	CS	60	47	3.63(2.29–5.76)	3.19(1.87–5.44)*	0.001
	Vaginal	78	222	1	1	
Multiple Pregnancy	Yes	27	15	4.12(2.11–8.05)	2.98(1.39–6.36)*	0.002
	No	111	254	1	1	
PROM	Yes	31	45	1.44(0.86–2.41)	1.17(0.64–2.15)	0.61
	No	107	224	1	1	
Presence of HIV	Yes	24	34	1.46(0.82–2.57)	1.28(0.66–2.47)	0.47
	No	114	235	1		
Length of Labor	≥24 hrs	66	110	1.33(0.88–2.00)	1.32(0.82–2.15)	0.26
	<24hr	72	159	1	1	
Gestational Age	<37 weeks of gestational age	46	32	3.70(2.22–6.18)	2.83(1.58–508)*	0.003
	≥37 week of gestational age	92	237	1	1	

## Discussion

Identifying predictors of stillbirth is fundamental to mitigate the problem and to help couples have a healthy baby. Though, few studies have been done to identify predictors of stillbirth, the most important predictors were not assessed. Therefore, this study assessed predictors of still birth by incorporating the most proximate factors affecting birth outcomes in the study setting. Of the variables that were assessed in this study; having no ANC visit, being hypertensive during pregnancy, having cesarean section mode of delivery, having multiple pregnancies and preterm birth had a significant association with stillbirth.

Mothers who had no ANC visits were almost four times at a higher risk of developing stillbirth than mothers who had ANC visits. This finding is consistent with those of studies conducted in Northern Ethiopia and Nepal [26, 27]. This could be because mothers who had no antenatal care visits will not receive any antenatal care services including risk assessment, care provision, and health promotion. Hence, mothers who had no ANC visits will not gain a screening opportunity for certain risk factors that are associated with antepartum hemorrhage, intrapartum hemorrhage, certain medical conditions, infection, or hypertensive disorder. If risks are found, health care providers will not have the opportunity to manage or treat specific conditions. Additionally, they could not provide counseling to mothers and families, all of which could not help to prevent stillbirth. This report also indicates that the provision of good and sufficient perinatal counseling in the MCH clinic will help to mitigate the problem of stillbirth.

The government of Ethiopia provides free antenatal care check-ups through public health facilities and has a standard protocol for antenatal check-ups [31, 32]. The 2019 EMDHS results show that 74% of women who gave birth in the 5 years preceding the survey received antenatal care from a skilled provider at least once during their last pregnancy. Four in 10 women (43%) had four or more ANC visits for their most recent live births [32]. Moreover, large disparities exist in the access to antenatal care by socioeconomic status, with women from rural residents and those with the least education have the lowest access to care in Ethiopia [32].

Hypertension during pregnancy is significantly associated with stillbirths. Mothers with hypertension during pregnancy were almost 3 times more likely to develop stillbirth compared to mothers who had no hypertension during pregnancy. This finding is congruent with those of studies conducted in Taiwan, Nepal, and England [28, 29, 35]. This is because mothers who have hypertension during pregnancy do not have sufficient blood flow to the placenta compared with mothers who have no hypertension during pregnancy. In addition to this, blood pressure falls by the second trimester in most cases but rises during the third trimester to a level somewhat above in the early pregnancy. Hence, the fetus will receive less oxygen and fewer nutrients, which in turn causes the fetus to be born a stillbirth.

Hypertensive pregnancies are responsible for 4–9% of all fetal deaths. The stillbirth rate is 5-52/ 1000 births, depending on the severity of complications from hypertension [36]. Hypertension is one of the most common medical conditions that complicate pregnancy which results in intrauterine growth retardation, intrauterine fetal death, and stillbirths [29].

Mothers who had delivered in the cesarean section were 3.2 times at a higher risk of developing stillbirth compared to their counterparts. This finding is congruent with a global survey conducted on stillbirths [37, 38]. Cesarean section carries many risks to women including blood loss, maternal inactivity, injury to organs, blood clot, death of the mother, increased risk of uterine rupture, and stillbirth. This finding suggests that health care providers should consider the potential risk of cesarean section while assessing the clinical indications for cesarean section. Cesarean section should be performed only when there is convincing clinical indications, in other words, cesarean section mode of delivery should not be considered for non-medical reasons.

Studies conducted in Denmark and West Africa also demonstrated that cesarean section is a predictor of stillbirth [39, 40]. This report implied that health caregivers working on MCH should take into consideration the possible risk of cesarean section while assessing the risk of pregnancy at the ANC clinic. The practice of cesarean section should be implemented only when there are compelling medical clues. This finding also showed that reducing the cesarean section mode of delivery could be a preventive strategy for the occurrence of stillbirth.

Mothers who had multiple pregnancies were 3 times at risk of stillbirth than mothers who had no multiple pregnancies. Studies conducted in 12 hospitals across Kenya, Malawi, Sierra Leone, Zimbabwe, and Jordan revealed that multiple pregnancies are a risk factor for the occurrence of stillbirth [13, 23]. Women with multiple pregnancies are more likely to develop preterm labor and birth, placental problems, gestational high blood pressure, gestational diabetes, preeclampsia, postpartum hemorrhage, anemia, and fetal complications including spinal Bifida, twin-to-twin transfusion syndrome, and other neural tube defects in the digestive tract and heart.

This health challenge often develops prematurely and is worse than that in gestation with one baby. It can also accelerate the early separation of the placenta (placental abruption). This in turn leads to fetal death (i.e., death before the complete expulsion or extraction of a product of conception from its mother) in the third trimester (≥28 completed weeks of gestation).

Women who had preterm birth were 3 times at a higher risk of stillbirth compared to women who had term birth. This finding is similar to those of studies conducted in England, India, and Southern Ethiopia [15, 20, 23]. A possible explanation could be that women with preterm birth are more likely to result in stillbirth which is mainly associated with trouble in breathing due to an immature respiratory system and experience prolonged pauses in their breathing, known as apnea, heart problems, temperature control problems including the absence of stored body fat of a full-term infants, and inability to generate enough heat to counteract what is lost through the surface of their bodies, immune system problems, and infection. This in turn causes stillbirth (a baby born with no signs of life at or after 28 weeks gestation).

The limitation of this study is that it does not incorporate some of the variables that are addressed in the community, such as wealth index, nutritional status, and cultural aspects. Therefore, other scholars should consider those situations, and it is also very important if they supplement or triangulate with a qualitative study to dig out untouched aspects. Thus, the readers should consider the limitations of this study while interpreting the finding, and the other scholars will do more to overcome those limitations. The finding of this study gives an overriding reputation to tackle factors determining stillbirth, which leads to stillbirth and predisposing factors for stillbirth.

## Conclusions

In this study we found that being hypertension during pregnancy, having cesarean section mode of delivery, having no ANC visits, having multiple pregnancies and preterm birth were independent risk factors associated with still birth. The predictors of stillbirth identified are manageable and can be amenable to interventions. Therefore, appropriate prevention strategies during antepartum and intrapartum care should be focused on to tackle these risk factors of stillbirth. Strengthening maternal antenatal care utilization should be encouraged by providing appropriate information for the mothers. There is a need to identify, screen, and critically follow high-risk mothers: those who had different complications during pregnancy, those who faced hypertension during pregnancy, those who identified as multiple pregnancies, and those undergoing cesarean section due to different indications.

## Supporting information

S1 Questionnaire English and Amharic version questionnaire. (DOCX)

S1 Dataset Minimal data set. (SAV)

## List of abbreviation

AOR: Adjusted odds ratio
ANC: Antenatal Care
PROM: Premature Rupture of Membrane
C/S: Cesarean section
WHO: World Health Organization
