# Supplementary material for: Predictors of stillbirth among women who had given birth in Southern Ethiopia, 2020: A case-control study
Source: PLoS One. 2021 May 3;16(5):e0249865. doi: 10.1371/journal.pone.0249865 (PMC8092801; doi:10.1371/journal.pone.0249865)
Supplement: S1 Questionnaire — (DOCX) [file pone.0249865.s001.docx]

**English Version Questionnaire**

**Interview Record for Quantitative Data Identification Number**

Questionnaire developed for predictors of stillbirth among women who had given birth in Southern Ethiopia, 2020:

| **S.No.** | **Question** | **Response** | **Skip** |
| --- | --- | --- | --- |
| **Part I: Socio- demographic Characteristics** | | | |
| 101 | Age of the mother | /_____________/ in complete year |  |
| 102 | Educational status of mother | 1. Cannot able to read and write 2. Can read and write 3. Grade 1-8 4. Grade 9-12 5. College and above |  |
| 103 | Marital status of the mother | 1. Married  2. Single  3. Divorced  4. Windowed  5. Other |  |
| 104 | Religion | 1. Orthodox 2. Catholic 3. Protestant 4. Other, Specify____________ |  |
| 105 | Occupation of mother | 1. House wife 2. Merchant 3. Government employer 4. Daily laborer 5. Other, Specify_____________ |  |
| 106 | Place of residence | 1. Urban 2. Rural |  |
| 107 | Average monthly income | /_______________/ in ETH birr |  |
| **Part II: Obstetrics and gynecology history of the women** | | | |
| 201 | Do you have ante natal care visit? | 1. Yes 2. No |  |
| 202 | If yes how many no of visit it was | __________ in number |  |
| 203 | Gestational Age | ­­­­­­­­­­­­­­­­­______________( in week) |  |
| 204 | Do you have prior history of Abortion | 1. Yes 2. No |  |
| 205 | Do you have prior history of stillbirth | - - - 1. Yes       2. No |  |
| 206 | Do you have HIV | 1. Yes 2. No |  |
| 207 | Do you have hypertension during Pregnancy | 1. Yes 2. No |  |
| 208 | How many times you gave live birth | ­­­­­­­­­­­­­­­­­­­­­­­­­­­­­­­­­­­­­­__________ |  |
| 209 | What was you mode of delivery | 1. Vagina 2. C/S |  |
| 210 | Do you have multiple pregnancy in this birth | 1. Yes 2. No |  |
| 211 | Had you faced any type of complication during the delivery? | 1. Yes 2. No |  |
| 212 | Do you have PROM | - - - 1. Yes       2. No |  |
| 213 | Presence of anemia | 1. Yes 2. No |  |
| 214 | How much your labor has taken during delivery | ____________ ( in Hour) |  |
| 215 | Where you gave birth for index child? | 1. My home 2. Health Center 3. Hospital 4. In mom home |  |
| 216 | Was the instrument used to cut cord boiled prior to use? | 1. Yes 2. No |  |
| **Part III: Fetal condition** | | | |
| 301 | Sex of your child? | 1. Male 2. Female |  |
| 302 | Weight of the new born (kg) | 1. _________(in Kg) |  |
| 303 | Fetal heart rate monitoring as per protocol? | 1. Yes 2. No |  |
| 304 | Use of partograph | 1. Yes 2. No |  |
| 305 | Feto maternal hemorrhage | 1. Yes 2. No |  |
| 306 | Fetal blood loss | 1. Yes 2. No |  |
| 307 | Presentation of fetus | 1. Vertex Presentation 2. Breach Presentation 3. Shoulder Presentation 4. Other, Specify___________ |  |

**Amharic Version Questionnaire**

| **S.No.** | **ጥያቄ** | **መልስ** | **ይዝለሉ** |
| --- | --- | --- | --- |
| ክፍል አንድ**:** የማህበራዊና ስነህዝባዊ ስብጥር መረጃ | | | |
| 101 | እድሜ | /_____________/ በአመት |  |
| 102 | የትምህርት ደረጃ | - - - 1. ማንበብ እና መጻፍ የማይችል       2. ማንበብ እና መጻፍ የሚይችል       3. ከ1-8ኛ ክፍል የተማረ       4. ከ9-12ኛ ክፍል የተማረ       5. ኮሌጂ እና ከዚያ በላይ |  |
| 103 | ሀይማኖት | - - - 1. ኦርቶዶክስ       2. ካቶሊክ       3. ፕሮቴስታት       4. ሌላ ካለ ይግለጹ_____________ |  |
| 104 | የትዳር ሁኔታ፦ | 1. ያገባች 2. ያላገባች 3. የተፋታች 4. ባሏየሞተባት 5. ሌላ ካለ ይግለጹ__________ |  |
| 105 | የስራ ሁኔታ | - - - 1. የቤት እመቤት       2. ነጋዴ       3. የመንግስት ሰራተኛ       4. የቀን ሰራተኛ       5. ሌላ ካለ ይግለጹ_____________ |  |
| 106 | የመኖሪያ አድራሻ | - - - 1. ከተማ       2. ገጠር |  |
| 107 | የቤተሰብ ወራዊ ገቢ | /_______________/ በብር |  |
| **ክፍል ሁለት: የማህጸን እና ፅንስ ታሪክን ይመለከታል** | | | |
| 201 | የርግዝና ከትትል ነበረዎት | 1. አወ 2. የለም |  |
| 202 | አወ ካሉ | ስንት ጊዜ ክትትል ነበረዎት________ |  |
| 203 | ካረገዙ ምን ያህል ጊዜ ይሆነዎታል | ­­­­­­­­­­­­­­­­­______________ በሳምንት |  |
| 204 | ጽንስ የማቋረጥ ታሪክ ነበረዎት | 1. አወ  2. የለም |  |
| 205 | የርግዝና ጊዜዎ 7 ወር (28 ሳምንት ከኋላ ሆነ ሙቶ የተወለደ ነበረ ወይ | 1. አወ 2. የለም |  |
| 206 | ኤ ች አይ ቪ በሽታ አለበወት | 1. አወ 2. የለም |  |
| 207 | የደም ግፊት ነበረዎት | 1. አወ 2. የለም |  |
| 208 | በህይዎት የተወለደ ልጂ ምን ያህል አለዎት | ­­­­­­­­­­­­­­­­­­­­­­­­­­­­­­­­­­­­­­__________ በቁጥር ይግለጹ |  |
| 209 | በምን አይነት የማዋለጂያ መንገድ ነበር የመጨረሻ ልጂዎትን የዎለዱት | 1. በማህጸን 2. በቀዶ ጥገና ዘዴ |  |
| 210 | የርግዝና ሁኔታ | 1. አንድ 2. መንታ እና ከዛ |  |
| 211 | በርግዝና ጊዜ ከባድ የሚባል የጤና ችግር አጋጥሞት ነበር | 1. አወ 2. የለም |  |
| 212 | ምጥ ከመጀርዎት በፊት የፈሰሰ ፈሳሽ ነገር ነበር | 1. አወ 2. የለም |  |
| 213 | የደም ማነስ ነበረበዎት | 1. አወ 2. የለም |  |
| 214 | የደም መፍሰስ ችግር ነበር | 1. አወ 2. የለም |  |
| 215 | ምጥዎ ምን ያህል ጊዜ ወሰደ | ____________ በስአት |  |
| 216 | የመጨረሻ ልጆዎን የትወለዱት | 1. ቤት 2. ጤና ጣቢያ 3. ሆስቢታል 4. ሌላ ካለ ይግለጽ |  |
| **ክፍል ሶስት: የህጻኑን ሁኔታ ይመለከታል** | | | |
| 301 | የህጻኑ ጾታ | 1. ወንድ 2. ሴት |  |
| 302 | የህጻኑ ክብደት | 1. _________ በኪሎ ገራም |  |
| 303 | የህጻኑ የልብ ምት ክትትል ነበረ | 1. አወ 2. የለም |  |
| 304 | የፓርቶግራፍ ክትትል ነበረ | - - - 1. አወ       2. የለም |  |
| 305 | ህጻኑ የደም በፍሰስ ችግር አጋጥሞት ነበር | 1. አወ 2. የለም |  |
| 306 | የህጻኑ እትብት ከመቆረጡ በፊት የትብት መቁረጫውን አፍልተውት ነበር ? | 1. አወ 2. የለም   የለም |  |
| 307 | የህጻኑ በማህጸን ውስጥ አቀማመጥ | - - - 1. በጭንቅላቱ       2. በቂጡ       3. በትክሻው       4. ሌላ ካለ ይግለጹ___________ |  |
